# Laparoscopic cholecystectomy in-patient with situs inversus

**DOI:** 10.4103/0972-9941.25674

**Published:** 2006-03

**Authors:** A Y Shah, B C Patel, B A Panchal

**Affiliations:** Parth Surgical Hospital, Shashi Complex, Near Swaminarayan Avenue, Anjali Cinema Cross Road, Vasana, Ahmedabad - 380007, Gujarat, India

**Keywords:** Situs inversus, laparoscopic cholecsytectomy

## Abstract

In modern era, laparoscopic surgery is gold standard for gall bladder calculi. Situs inversus is a rare condition. To diagnose as well as operate any pathology in such patients is difficult. Laparoscopic cholecystectomy in such patient is a challenge but not contraindication.

## INTRODUCTION

In 1600, Fabricius reported the first known case of situs inversus in humans.[1] The incidence is thought to be in the region of 1:5000 to 1:20000.[1] It may be partial, where the transposition is confined to either the abdominal or the thoracic viscera, or complete, i.e, involving both the cavities.[2] It is associated with a number of other conditions such as Kartagener's syndrome (bronchiectasis, sinusitis, situs inversus) and cardiac anomalies. There is no current evidence that situs inversus predisposes to cholelithiasis.[1] Change in anatomical disposition of organs not only influences the location of symptoms and signs arising from a diseased organ but also impose special demands on the diagnostic and surgical skills of the surgeon. Since Mouret first performed it in 1987, laparoscopic cholecystectomy has become the standard operative procedure for gallbladder disease. It is associated with reduced hospital stay, fewer respiratory complications, less pain and a faster return to work. We report a patient with complete situs inversus who presented with biliary colic and underwent laparoscopic cholecystectomy.

## CASE REPORT

**Figure 1:**
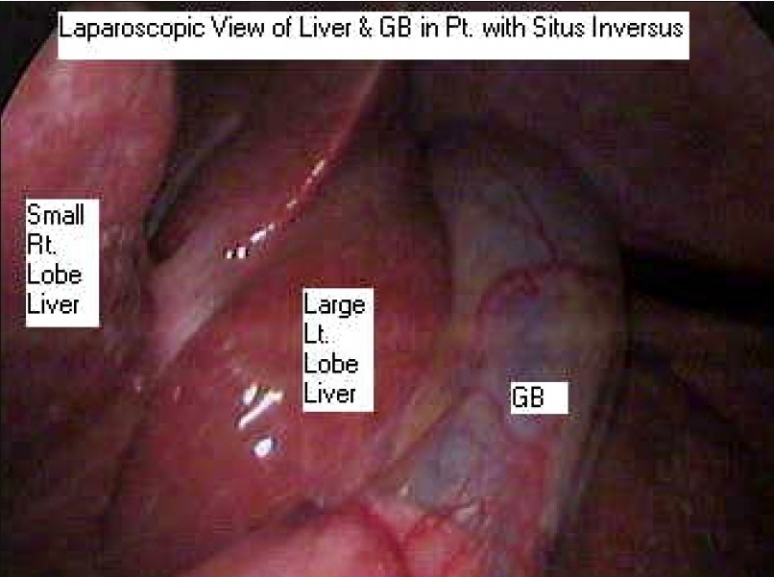
Laparoscopic View of GB in patient with situs inversus

A 60-year old female presented with complain of recurrent colicky left upper abdominal pain associated with nausea, vomiting and flatulent dyspepsia for 2–3 months. There was no history of fever, jaundice or hospitalization. Abdominal examination was normal. On chest examination apex beat found on right side of chest. Liver function tests were normal. Ultrasonography of the abdomen revealed situs inversus of all visceral structures [Figures 1]. The liver was located on the left, had normal echotexture and showed no evidence of intrahepatic biliary dilatation. The gall bladder was located on the left side, was contracted and contained multiple calculi. Common bile duct was normal. Chest X-ray screening showed the heart to be on the right side. This suggested the possibility of this patient with situs inversus was suffering from gallstone colic. A laparoscopic cholecystectomy was planned. Laparoscopic cholecystectomy was undertaken using the standard 4-port technique. The surgical team changed sides with the primary surgeon and first assistant on the patients right and the second assistant on the left. The video monitor was placed near head of patient on left side. Pneumoperitoneum was created to a pressure of 14 mmHg using a Veress' needle through an umbilical incision and the 0-degree telescope was inserted through a 10-mm umbilical (primary) port (Port 1). A 5-mm port was placed just below to left costal cartilage in anterior axillary line and a grasper was inserted to catch and retract fundus of the gall bladder (Port 2). A 10 mm port was inserted 4 cm below xiphoid 1 cm right to midline (Port 3) and another 5 mm port was inserted 5 cm below left costal cartilage in left midclavicular line (Port 4). Being a right-handed person, it was difficult to dissect though port 3 as been done routinely. We used port 3 to grasp neck of GB whereas port 4 for dissection. The neck of the gallbladder was grapsed with a tissue forceps introduced from port 3. Ultrasonic shears were used to dissect omental adhesions covering the Calot's triangle. Next, with the help of ultrasonic dissection the cystic artery and duct were dissected. Clips were applied to the cystic artery and duct which were divided. The gallbladder neck was grasped with a tissue grasper introduced from port 3 and the gall bladder was dissected from the fossa with a hook passed from port 4. The gallbladder was removed from port 3 and a tube drain was placed in the subhepatic space. The procedure was uneventful and the patient recovered smoothly.

## DISCUSSION

Situs inversus occurs once in approximately 20,000 live births and has an autosomal recessive inheritance.[1] The frequency of cholelithiasis in patients with situs inversus is similar to that in the general population.[2] However, the condition may present diagnostic difficulty. Pain of biliary colic may be located in the epigastrium or in the left subcostal region and that of cholecystitis radiates to the left infrascapular region and the left shoulder.

In patients with situs inversus, the mirror image anatomy poses difficulty in orientation during laparoscopic cholecystectomy. First, various laparoscopy ports need to be positioned at sites that are mirror image of those in the usual patient. Second, the surgeon needs to reorient visual images and surgical steps in an anatomical field that has undergone clockwise rotation. In the reports described previously the surgeon dissected with his left hand or asked for assistance to grasp the neck of the gall bladder. But we changed the conventional technique and the port used for grasping the neck was used for dissection and vise versa.[1,3] Though laparoscopic cholecystectomy in such patients is technically more demanding, an experienced laparoscopic surgeon can perform it safely. Thus, situs inversus totalis does not appear to be contraindication to laparoscopic cholecystectomy.[4]

## References

[1] McKay D, Blake G (2005). Laparoscopic cholecystectomy in situs inversus totalis:a case report. BMC Surg.

[2] Wood GO, Blalock A (1940). Situs inversus totalis and disease of the biliary tract. Arch Surg.

[3] Takei HT, Maxwell JG, Clancy TV, Tinsley EA (1992). Laparoscopic cholecystectomy in situs inversus totalis. J Laparoendosc Surg.

[4] Banerjee JS, Vyas FL, Jesudason MR, Govil S, Muthusami JC (2004). Laparoscopic cholecystectomy in a patient with situs inversus. Indian J Gastroenterol.

